# Supplementary material for: Towards stronger antenatal care: Understanding predictors of late presentation to antenatal services and implications for obstetric risk management in Rwanda
Source: PLoS One. 2021 Aug 25;16(8):e0256415. doi: 10.1371/journal.pone.0256415 (PMC8386859; doi:10.1371/journal.pone.0256415)
Supplement: S1 File — (DOCX) [file pone.0256415.s001.docx]

**Participant Enrollment Survey**

1. Akarere *District*

2. Ikigo nderabuzima? *Health center?*

3. Study ID (FULL) *Full Study ID (nine-digit number)*

4. Imyaka y'amavuko *Age*

5. Umudugudu *Village*

6. Itariki yatangiriyeho *Date of enrollment*

7. Ese kuva utwite iyi nda waba warigeze kwiyambaza umujyanama w'ubuzima wo mu mudugudu wawe mbere yo kuza ku kigo nderabuzima? *Did you have any contact with the Community Health Worker in your village about this pregnancy before coming to the health center?*

8. Ese uwo mujyanama w'ubuzima yagufashe ikizamini cy'inkari? *Did the CHW conduct a urine pregnancy test?*

9. Wize amashuri angahe? 0, Nta na rimwe, 1, Nize abanza sinayarangiza, 2, Nize abanza ndayarangiza, 3, Nize ayisumbuye sinayarangiza, 4, Nize ayisumbuye ndayarangiza, 5, Nize amashuri makuru/kaminuza sinabirangiza, 6, Nize amashuri makuru/kaminuza ndabirangiza *What is the highest level of education you have completed? 0, None 1, Some primary 2, Completed primary 3, Some secondary 4, Completed secondary 5, Some college/university 6, Completed college/university*

10. Ukora iki? *What is your occupation?*

11. Ese hari amafaranga ukorera winjiza mu rugo? *Do you earn money for your household?*

12. *Mu kwezi gushize, ... In the last month, ...*

12a. …ese hari igihe wigeze uhangayika ufite ubwoba bw'uko ibiribwa byashoboraga gushira mbere y'uko mubona amafaranga yo kugura? …*have you ever been worried that the food would run out before you had money to buy more?*

12b. …ese ibiribwa mwaguze byamaze igihe gito maze mubura amafaranga yo kugura ibindi? …*has the food you bought not lasted and you didn’t have money to buy more?*

13. Ese waba unywa itabi ry'ibibabi/isigara? *Do you smoke tobacco/cigarettes?*

14. Ese waba unywa ibinyobwa bisembuye? *Do you drink alcohol?*

15. Ese wumva ushobora kuganira n'umugabo mubana ukamubwira ikintu cyose wifuzako yamenya ku birebana n'itwita ryawe? *Do you feel you can discuss any matter related to your pregnancy openly with your partner?*

16. Ese ibintu bikurikira byigeze bikubaho: A, Kubyara hasigaye igihe kirenga ukwezi kugira ngo itariki nyayo y'ivuka yari iteganyijwe igere? | B, Umwana yavutse afite hasi y'ibiro 2.5 | C, Umwana yavutse apfuye, cyangwa yapfuye mu masaha 24 akurikira ivuka rye | D, Umwana yapfuye mu minsi 28 ikurikira ivuka rye | E, Kuvamo kw'inda cyangwa kuyikuramo | Ntanakimwe muri ibi bisubizo.  *Do you have any prior history of: A, Giving birth more than one month before you were due? | B, Baby born less than 2.5kg | C, Stillbirth, or baby dying within first 24 hours | D, Baby died within first 28 days of life | E, Abortion or early termination of pregnancy | None of the above responses*

17. Ese ubwo uheruka gutwita wipimishije kangahe? *In your last pregnancy, how many times did you attend ANC?*
