# Supplementary material for: Towards stronger antenatal care: Understanding predictors of late presentation to antenatal services and implications for obstetric risk management in Rwanda
Source: PLoS One. 2021 Aug 25;16(8):e0256415. doi: 10.1371/journal.pone.0256415 (PMC8386859; doi:10.1371/journal.pone.0256415)
Supplement: Attachment — Submitted filename: Plos.docx [file pone.0256415.s002.docx]

1. Abstract

- On line 41‘’Earning income’’: not clear
- Line 48, use conclusion instead of discussion

2. Introduction

- On page 6 line 112 ‘‘this analysis identified predictors of late (≥16 weeks GA)…’’. Why did you use 16 weeks of gestational age as a cut-off pint? WHO recommends pregnant women have to start their first ANC booking within the first 12 weeks of gestational age. See the following references.
- *World Health Organization. WHO recommendations on antenatal care for a positive pregnancy experience [Internet]. Geneva, Switzerland; 2016. Available from: ttps://apps.who.int/iris/bitstream/10665/250796/.../9789241549912-eng.pdf*
- *Tuncalp Ӧ, Pena-Rosas J, Lawrie T, Bucagu M, Oladapo O, Portela A, et al. WHO recommendations on antenatal care for a positive pregnancy experience — going beyond survival. BJOG. 2017;124:860–2.*

3. Methods

- From line 132-133, ‘‘only participants with completed ANC care records were included in the final analysis.’’ If this is the case, is it representative?

4. **Analysis**

- Data analysis is not well addressed and robust
- From line 165-166, ‘‘Chi-squared tests were performed to identify variables

significantly associated with late presentation to ANC.’’ why you preferred Chi-squared tests than odds ratios?

- Not clear on the selection of measures of association used. For instance, Chi-squared tests vs. Odds ratios.

5. Ethical consideration

- Line 198, ‘‘Written consent was obtained from all participants.’’ How did you secure it? Are all read and write to give you written consent?

6. Discussion

- Lacks author argument

7. Limitations

- Line 370, ‘‘we found that many clinical variables had poor quality.’’ many clinical variables specifically………had poor quality
