# Supplementary material for: Towards stronger antenatal care: Understanding predictors of late presentation to antenatal services and implications for obstetric risk management in Rwanda
Source: PLoS One. 2021 Aug 25;16(8):e0256415. doi: 10.1371/journal.pone.0256415 (PMC8386859; doi:10.1371/journal.pone.0256415)
Supplement: Attachment — Submitted filename: Comments to Authors Reviewer 5.docx [file pone.0256415.s003.docx]

## Title:

Towards stronger antenatal care: understanding predictors of late presentation to antenatal services and implications for obstetric risk management in Rwanda

Journal: International Journal of Health Promotion

**Manuscript ID**: ONE-D-20-22930

Manuscript type: Research Article

**Date:** 5 November 2020

**Reviewer's report:**

Initiating antenatal care (ANC) in the first trimester and identifying obstetric risk factors early are crucial for improving birth outcomes. Also, service providers’ knowledge of predictors of initiation of ANC has implications for obstetric risk management. This can help improve quality of care. However, English language needs some input; a few have provided.

# **General comments**

1. The abstract convey what is in the manuscript
2. The data is sound.
3. The method section of the manuscript requires a major revision
4. The tables are genuine and well described
5. Some parts of the discussions needs revision to situate especially contrasting results in academic discourse
6. Conclusions reflect the results

I thus, recommend that the manuscript be accepted and the authors asked to revise the manuscript before publication.

# Abstract

Word count =306

1. The title: Towards stronger antenatal care: understanding predictors of late presentation to antenatal services and implications for obstetric risk management in Rwanda

The title needs revision to cover early initiation of ANC (<16 weeks) since it is part of the analysis as shown in Table 1 and the conclusion

**Introduction**

1. P. 5, line 89: insert ‘as’ for the sentence read such as the practice of disclosing pregnancy after the second trimester.
2. P. 5, line 90-91: I suggest ‘poor experiences’ be replaced with ‘negative experiences’
3. P. 7, line 119-121: The authors should avoid repeating words sentences. The second ‘how’ in the sentence. I suggest: …, and inform messages tailored to multiparious and nulliparous women.
4. P. 7, 133: … completed ANC care… the ‘care’ is repetition and should be deleted.

# **Results**

5. P. 10, line 203 and 206-208: By conversion, a sentence should not begin with a figure. I suggest the sentences should be re-written.
6. P. 15 and 16, Tables 1, 2, 3 and 4 and 5: The ‘p-values’. The ‘p’ should be capitalised as ‘P-values’
7. P. 16: Idetified is not necessary in Table 5
8. All the information in brackets in Tables 1, 2, 3 and 4 (≥ 16 weeks GA) and very late (≥ 24 weeks GA) is not necessary. They are already difined in the text.
9. I expect the authors to emphasise finding regarding the benefits of initiating ANC < 16 weeks.

# **Discussion**

10. P. 17, line 276: Despite ongoing to increase early ANC attendance, that the .... ‘that’ should be deleted.
11. P. 17, 283-287: I suggest the authors look for evidence to explain and support their assertion that contribution to household income was a stronger predictor of delayed ANC-1. Also, could you please emphasise what your study adds?
12. P. 18, line 295: More effective strategy increase .... There should be ‘to’ as: ... strategy to increase ...
13. P. 18, line 300-302: However, barriers to continuing ANC care may emerge for women for whom ANC-1. The sentence should be: However, barriers to continuing ANC care may emerge for women for whom ANC-1. I suggest ‘ANC’ be deleted and ‘for whom’ replaced with ‘whose’
14. P. 19, line 333: why put ‘none’ into brackets. The sentence clear without ‘none’
15. P. 20, line 342: Despite conducting data strengthening activities, our investgation found lower than expected rates of obstetric risk factors were identified in the study population overall. ‘were identified’ is a repetition so need to be deleted.
16. P. 20, line 345-346: The sentence needs to be re-written to make comprehension easy for readers. I suggest: Other important screening results including diabetes and syphilis were systematically missing from the health record and thus were excluded from our final analysis.
17. P. 20, line 351: ... function of ANC care. ‘care’ is repetition and should be deleted.
18. P. 20, line 357: Delete the second ‘in’ in the sentence. i.e. This study also reported large gaps in provider knowledge, including the ability to …

**Conclusion**

19. The conclusion should also emphasise the benefits and predictors of early ANC attendance to inform interventions.
20. The conclusion early intervention is too unspecific as this vary in different cultures
21. The recommendation that community-based pregnancy testing could increase early pregnancy discovery and prompt earlier attendance at ANC clinics can be contested as the data does not show that late attendance is attributed to not being aware of the pregnancy.
