# Supplementary material for: Towards stronger antenatal care: Understanding predictors of late presentation to antenatal services and implications for obstetric risk management in Rwanda
Source: PLoS One. 2021 Aug 25;16(8):e0256415. doi: 10.1371/journal.pone.0256415 (PMC8386859; doi:10.1371/journal.pone.0256415)
Supplement: Attachment — Submitted filename: Response to reviewers.docx [file pone.0256415.s004.docx]

December 27, 2020

Dr. Hanna Landenmark

Associate Editor, PLOS ONE

Dear Dr. Landenmark,

On behalf of my coauthors, I would like to thank you for the opportunity to revise and resubmit our manuscript PONE-D-20-22930, entitled “Towards stronger antenatal care: Understanding predictors of late presentation to antenatal services and implications for obstetric risk management in Rwanda”

We found the reviewers’ comments to be very helpful in revising the manuscript and have carefully considered and responded to each suggestion. In the majority of cases we were successful in incorporating the reviewers’ feedback into our revised manuscript. At the end of this letter, we have included a response to reviewers in which we address each comment the reviewers made. In our response to reviewers, the reviewers’ comments are numbered, and our responses follow below prefaced by “Author response.” Corresponding changes are highlighted in the manuscript text in the revised file. We have also addressed the additional editorial requirements and attached our response to the end of this document.

Thank you again for your consideration of our revised manuscript. We look forward to hearing from you in due time regarding our submission and to respond to any further questions and comments you may have.

Sincerely,

Christina Schmidt (corresponding author)

Doctor of Medicine Candidate, School of Medicine

University of California San Francisco

Email: [christina.schmidt@ucsf.edu](mailto:christina.schmidt@ucsf.edu)

Phone: +1 503 703 4953

**Response to Reviewer #1 comments:**

A secondary analysis was conducted with the aim of identifying factors associated with delayed initiation of antenatal care (ANC) and comparing the risks between those presenting early and late for care. Adjusting for clusters, backwards stepwise regression analysis were used to identify predictors of first ANC visit. The statistical methods are vague; therefore, the conclusions are unclear.

**R1.1: It seems that either logistic regression models should be used when predicting the dichotomies of ≥ 16 weeks or ≥ 24 weeks or linear regression models to predict the continuous value for week of ANC-1. Additionally, in the backward selection model it is common to include candidate predictors with univariate p-values ≤ 0.10.**

Author response: Thank you for this feedback. We have adjusted our backward elimination models to include candidate predictors with univariate p-values <0.20. We selected a cutoff of <0.20 based on suggestions from other reviewers, and evidence that backward elimination procedures with mild significance level criterion are superior (we have included citations in our revised draft).

To provide additional clarity, we described our backwards elimination process in greater detail, including more explicitly stating how variables were evaluated and removed to reach our reduced models. We also revised Table 3 to include both the full and reduced multivariable models. Finally, we clarified that we used logistic regression with cluster robust standard errors to account for the clustering effects of health facilities. Please see lines 182-192 for an updated description of our statistical methods incorporating these changes. We have updated all tables in our manuscript to reflect the changes that we made to our analysis.

**R1.2: Chi-square tests are used to show association. Use logistic or linear regression models for prediction, even for univariate tests.**

Author response: We have adjusted our analysis to use logistic regression models for our univariate tests.

**Response to Reviewer #2 comments:**

**Abstract**

**R2.1: The results are confusing. The study set out to assess factors associated with delayed initiation of the first antenatal visit, in lines 31-33. However, it is reporting factors associated with early initiation of antenatal care in part of the results section, rather than factors associated with late initiation of antenatal care, in lines 41-47.**

**• Educational attainment, prior spontaneous abortion and pregnancy testing were associated with earlier ANC-1 (p<0.001).**

**• Women with a prior preterm (p=0.024) or low birthweight (p=0.023) delivery were more likely to present before 16 weeks GA, while history of stillbirth or neonatal death were not associated with timing of ANC-1.**

**• Women presenting before 16 weeks GA were more likely to have no obstetric risk factors identified at ANC-1 (p<0.001).**

Author response: Thank you for this feedback. We have reframed our results in both the abstract and main manuscript to consistently discuss factors associated with delayed ANC-1, rather than reporting factors associated with both late and early initiation of care (see lines 42-48 for abstract revisions).

**R2.2: In lines 49-54, the authors present a discussion, but do not have a clear conclusion. Also, the authors do not indicate how awareness of the importance of antenatal care would be increased. They also do not indicate how health system strengthening may be done to identify the suggested risk factors. They do not seem to have a conclusion. They state:**

**• Outreach efforts should focus on increasing mothers’ awareness of the benefits of ANC and emphasize the importance of early identification and management of pregnancy-related complications.**

**• While fewer risk factors were identified among women presenting less than 16 weeks, lower than expected rates were identified in the study population overall.**

Author response: Thank you for this feedback. We have worked on strengthening our argument and conclusions both in our abstract and in the discussion section of our manuscript. In our revised draft, we situate these results in the context of Rwanda’s existing Community Health Worker outreach program. Please see lines 50-56 for our updated conclusion section of our abstract. In our revised discussion we further elaborate on the practical implications of the results of our analysis.

**Main article**

**R2.3: In lines 65-71, the authors state that many of the complications that lead to adverse outcomes can be maternal or neonatal outcomes can be prevented through early antenatal care, and mention hemorrhage as one of the factors, yet antenatal care may not mitigate hemorrhage-related maternal morbidity and mortality. Similarly, they suggest that antenatal care may reduce infection-related neonatal morbidity and mortality without any suggestion of how this can be achieved. This to me seems speculative, considering that most neonatal infection follow peripartum or postnatal complications.**

Author response: Thank you for this feedback. We have adjusted these sentences to more accurately and clearly capture the benefits of ANC for mother and neonates, including removing references to hemorrhage and neonatal infections.

**R2.4: In lines 84-93, the authors should provide an explanation of how many of the suggested factors lead to late presentation of antenatal care.**

Author response: We have added additional sentences outlining potential reasons why the various factors reported in the literature may lead to late presentation to ANC (see lines 91-92, 102).

**Methods**

**R2.5: In lines 158-162, the authors need to indicate the definitions used for anemia, hypertension, proteinuria and diabetes, as well as how these variables were measured. Is it present/absent or a definite cut off?**

Author response: These variables were directly abstracted from the standard maternity registers used at all prenatal health centers across Rwanda. We have added the following sentence to clarify: “In Rwanda, anemia, proteinuria, hypertension, multiple births, MUAC <21cm, diabetes, and syphilis are universally screened for at ANC-1 and noted as either present or absent in the maternity register using tick boxes.”

**R2.6: In lines 171-178, the data analysis conducted is not clear. At analysis, the authors state:**

**Variables that were significantly associated with delayed presentation to ANC at the = 0.05 level were retained for multivariable model building. This is too strict and erroneous, as they should have included all variables with a p-value of less than 0.2 or even those with clinical significance, such as complications in a prior pregnancy. The backward and stepwise method of variable selection is also not clear.**

Author response: We have made adjustments to our statistical analyses, including changing our p-value threshold to <0.2. We also expanded our discussion of our variable selection process to provide additional clarity. Please refer to reviewer comment R1.1, where we describe these revisions in greater detail.

**Discussion**

**R2.7: The discussion does not provide an explanation of how the suggested factors operate, for example educational attainement, parity, prior complications etc.**

Author response: Thank you for this feedback. We have worked to make our results more robust and include further explanations as to how these predictors may operate. This includes adding additional citations in the second paragraph of the discussion (addressing educational attainment and age), and more explicitly addressing parity and a history of prior obstetric complications in the fourth paragraph of the discussion.

**R2.8: It is also not clear how parity as a moderator was assesed, when you view the results presented, as the stratified analysies compared primiparous and multiparous, rather than parity as an independent variable.**

Author response: We have revised our description of our moderator analysis in lines 205-208: “To assess whether parity was a moderator of the relationship between GA at ANC-1 and each of the obstetric risk factors identified at ANC-1, logistic regression models with interaction terms were used (α=0.05).” We did not include a table/figure with these results, but state in lines 285-287 “Parity was not found to be a moderator of the relationship between GA at presentation to care and any of the obstetric risk factors identified at ANC-1 in our moderator analyses.” We hope that the adjustments that we made to these statements clarifies our methods.

**R2.9: In lines 331-335, the authors state that: This investigation also examined differences in the obstetric risk factors identified at participants’ first ANC visit. Women presenting before 16 weeks GA were more likely to have no identified risk factors (“none”). This may be partially due to the fact that some obstetric risk factors, such as multiple births or hypertension, may be identified at a higher rate at later gestational ages. However, the focus of their stated objective was to assess factors associated with late antenatal attendance. Also, their explanation of of lack of identifiable risk factor is not clear to me.**

Author response: We have re-written this paragraph of the manuscript to provide additional clarity. We now emphasize that while patients may go on to develop obstetric risk factors as they progress through pregnancy, early presentation to care allows for timely identification of new obstetric risks. Please see lines 374-386 for our revisions.

**R2.10 The authors add, in lines 338-340, that: multiparous patients do not appear to be at lower risk of pregnancy-related complications. Multiparous women should be encouraged to continue to attend ANC, due to the risk of delayed identification of modifiable risk factors. This also is not clear to me.**

Author response: Thank you for this feedback. We have attempted to clarify this point in our revisions and have added the following statement: “In the setting of emerging evidence that multiparous women may delay ANC due to the belief that their pregnancies are lower-risk, these results raise concern that delays in care among multiparous patients may result in later identification of important obstetrical risk factors.” Please see lines 374-386 for our revisions to this section.

**R2.11: The authors should comment, in the discussion, on the quality of antenatal care, that is activities conducted per visit, rather than the number and timing of antenatal care visits, and the influence of this on pregnancy outcomes (maternal and neonatal outcomes).**

Author response: We appreciate the importance of this point. In lines 397-409 we discuss the impact of the content of ANC visits on pregnancy outcomes and suggest that improving quality of care rather than increasing the number of visits may have a greater impact on pregnancy outcomes. We have restructured a few of the sentences in the paragraph to help clarify our main points.

**Response to Reviewer #3 comments:**

**1. Abstract**

**R3.1: On line 41‘’Earning income’’: not clear**

Author response: We have updated this phrase to say, “contributing to household income.”

**R3.2: Line 48, use conclusion instead of discussion**

Author response: Thank you for this feedback. We have made this change.

**2. Introduction**

**R3.3: On page 6 line 112 ‘‘this analysis identified predictors of late (≥16 weeks GA)…’’. Why did you use 16 weeks of gestational age as a cut-off pint? WHO recommends pregnant women have to start their first ANC booking within the first 12 weeks of gestational age. See the following references.**

**World Health Organization. WHO recommendations on antenatal care for a positive pregnancy experience [Internet]. Geneva, Switzerland; 2016. Available from: ttps://apps.who.int/iris/bitstream/10665/250796/.../9789241549912-eng.pdf**

**Tuncalp Ӧ, Pena-Rosas J, Lawrie T, Bucagu M, Oladapo O, Portela A, et al. WHO recommendations on antenatal care for a positive pregnancy experience — going beyond survival. BJOG. 2017;124:860–2.**

Author response: We selected 16 weeks as our cutoff for “late” presentation, as this is the definition used by the Rwandan Ministry of Health in their prenatal care performance indicators. The Rwanda Demographic Health Survey tracks the proportion of women who present before 16 weeks gestational age, and this indicator is used in the country’s results-based financing program for health facilities providing prenatal care. We have clarified the rationale behind selecting 16 weeks in our revised manuscript (both in the introduction and methods sections) and have included the following reference:

Assaf, Shireen, Sarah Staveteig, and Francine Birungi. 2018. Trends in Maternal Health in Rwanda: Further Analysis of the 2014-15 Demographic and Health Survey. DHS Further Analysis Reports No. 108. Rockville, Maryland, USA: ICF.

**3. Methods**

**R3.4: From line 132-133, ‘‘only participants with completed ANC care records were included in the final analysis.’’ If this is the case, is it representative?**

Author response: We assessed for differences between patients with complete and incomplete ANC records and found no significant difference in key sociodemographic characteristics. We have added the following sentence to our manuscript: “There were no significant differences in the sociodemographic characteristics of those with incomplete ANC records, as determined by linear models of key variables.”

**4. Analysis**

**R3.5: Data analysis is not well addressed and robust**

**From line 165-166, ‘‘Chi-squared tests were performed to identify variables significantly associated with late presentation to ANC.’’ why you preferred Chi-squared tests than odds ratios? Not clear on the selection of measures of association used. For instance, Chi-squared tests vs. Odds ratios.**

Author response: Thank you for this suggestion. We have adjusted our analysis to use logistic regression models for our univariate tests. We have also made adjustments to our multivariable analysis, which are described in our response to reviewer comment R1.1.

**R3.6: 5. Ethical consideration**

**Line 198, ‘‘Written consent was obtained from all participants.’’ How did you secure it? Are all read and write to give you written consent?**

Author response: Thank you for this feedback. We have clarified the process of obtaining consent for participants who were unable to read in our revised manuscript:

“All consent forms were translated into Kinyarwanda. Participants provided consent by reading and signing the consent form. For participants who were illiterate, a member of the study team verbally read the consent form in the presence of a witness and both the consented participant and witness signed the consent.”

**R3.7: 6. Discussion**

**Lacks author argument**

Author response: Thank you for this feedback. We have made changes to our discussion section to more clearly emphasize the public health importance of this study. We have added more explicit policy recommendations based on our results, and more clearly emphasize the importance of targeting interventions towards those at greatest risk of delaying ANC. We discuss how the insights produced by this study could be integrated into the country’s existing CHW program with the goal of facilitating earlier uptake of ANC services. We also discuss the contribution of our work to the ongoing debate surrounding the optimal number of ANC visits, suggesting that current screening and documentation at ANC-1 is inadequate. We argue that patients may be better served by strengthening the content of existing visits, rather than adding additional prenatal contacts.

**R3.8: 7. Limitations**

**Line 370, ‘‘we found that many clinical variables had poor quality.’’ many clinical variables specifically………had poor quality**

Author response: We have clarified that diabetes, syphilis and malaria were not systematically recorded in our dataset and were thus excluded from our analysis.

**Response to Reviewer #4 comments:**

Towards stronger antenatal care: understanding predictors of late

presentation to antenatal services and implications for obstetric risk

management in Rwanda

This study is of Public health relevance. The authors identified factors associated with delayed initiation of ANC and describe differences in the obstetric risks identified at the first ANC (ANC-1) visit between women presenting early and late to care.

I have the following comments

**R4.1: 1. L36 & L172…authors claimed to have used “Cluster-robust standard error models”. This is a model estimation method and not a model in itself. In essence, you used logistic regression and controlled for the clustering effect of the health centres**

Author response: Thank you for this feedback. In the manuscript we have clarified that we used logistic regression for our multivariable models and used cluster robust standard errors to account for the clustering effects of healthcare centers.

**R4.2: 2. What was the level of significance adopted?**

Author response: In our revised analysis, we used a univariate p-value cutoff of <0.2 for inclusion of variables in our multivariable models (please refer to reviewer comment R.1.1). We considered predictors in our final multivariable model to be statistically significant if they had a p-value <0.05.

**R4.3: 3. What was the main study outcome and variables explored?**

Author response: In the methods section, we have clarified that, “The primary outcomes in this analysis were late and very late presentation to ANC. Late presentation was defined as ≥16 weeks GA in accordance with the Rwandan Ministry of Health’s prenatal care performance indicators, and very late was defined as ≥24 weeks GA” (see line 173-175). We describe the predictors variables that we explored in the paragraph that followssecon. We have also clarified that “The secondary outcomes of interest in this analysis were the obstetric risk factors identified at ANC-1” (see line 198).

**R4.4: 4. L127 …what were the controls**

Author response: Thank you for raising this question. In our backward elimination process we retained age despite the fact that it was not significant, as we considered this be a potential confounder.

**R4.5: 5. What is the rationale for grouping women aged 20 to 34 together? This is a critical age group where most pregnancy occurred. The behaviour of young adults (20-24 years) are very distinct from those aged 25-34 years. Authors should reanalyse.**

Author response: Thank you for this suggestion. We initially grouped women aged 20-34 together to capture extremes of maternal age, but upon further consideration we agree with your suggestion. We have broken down age into the following categories in our revised analysis: 15-19, 20-24, 25-29, 30-34, 35+.

**R4.6: 6. Important variables such as Type of marriage (monogamy/ polygamy) was, autonomy, decision making power by the women were excluded**

Author response: Thank you for this feedback. Unfortunately, marital status was not collected in the primary dataset, and thus we were unable to include this in our secondary analysis. We have added this to our limitations section.

**R4.7: 7. Table 3: Why present both coefficient estimates and odds ratio? Delete the two columns on “Estimate (SE)”. It is redundant.**

Author response: We have adapted the tables in the manuscript after adjusting our analysis, and no longer report coefficient estimates.

**R4.8: 8. Did you adjust for multicollinearity among the explanatory variables?**

Author response: We assessed for multicollinearity using variance inflation factor and considered variables with VIF >2.5 to be collinear. For variables that were collinear (gravidity and parity), we included the variable more strongly associated with our outcome of interest. We have clarified this process in our revised manuscript in lines 183-185.

**R4.9: 9. Did authors assess the relationship between individual explanatory variables before choosing them as candidate variables in the multiple regression model?**

Author response: Thank you for raising this important point. Please see our response to R4.8.

**R4.10: 10. It is usual to first do this assessment (bivariate regression) and include only the variables that were significant at a specified level (say 10 or 20%) before you run the multiple regression. The outputs of the bivariate regression are the odds ratio, sometimes called crude odds ratio (COR) while those from the multiple regression are called adjusted odds ratio (AOR)**

Author response: Thank you for this feedback. We have made changes to our analysis based on this feedback and comments from other reviewers. Please refer to our response to reviewer comment R1.1, where we outline the changes that we have made to our analysis.

**R4.11: 11. Tables 4 and 5 should precede Table 3**

Author response: In our revised manuscript, we have attempted to clarify that in this analysis we had two separate goals: 1) to identify predictors and late presentation to ANC, and 2) to look for differences the obstetric risk factors identified at ANC-1 between women presenting early and late to care. Tables 4 and 5 address our second goal, and describe the obstetric risk factors identified once patients present to ANC. Given that these are clinical risk factors that are identified at ANC-1, we did not consider them to be predictors of delayed presentation to care and did not include them in the first part of our analysis. We hesitate to change our analysis given that our goal in producing of Tables 4 and 5 was to describe obstetric risk factors, and that other reviewers did not share similar feedback. We appreciate that this might have been confusing and have added additional subheadings to clarify this distinction.

**R4.12: 12. Why were the risk factors in Tables 4 and 5 excluded from the regression model in Table 3? Were they insignificant?**

Author response: Thank you for this question. Please see our response to comment R4.11, where we describe our goal in producing Tables 4 and 5.

**R4.813: 13. There are typos and grammar issues**

Author response: Thank you for noting these errors. We have addressed all typos and grammatical errors that we were able to identify on our review.

**R4.814: 14. Authors should make the public health importance of this study clearer**

Thank you for this feedback. We have made changes to our discussion section to more clearly emphasize the public health importance of this study. We have added more explicit policy recommendations based on our results, and more clearly emphasize the importance of targeting interventions towards those at greatest risk of delaying ANC. We discuss how the insights produced by this study could be integrated into the country’s existing CHW program with the goal of facilitating earlier uptake of ANC services. We also discuss the contribution of our work to the ongoing debate surrounding the optimal number of ANC visits, suggesting that current screening and documentation at ANC-1 is inadequate. We argue that patients may be better served by strengthening the content of existing visits, rather than adding additional prenatal contacts. This analysis was requested by members of the Rwandan Ministry of Health’s division of Maternal, Newborn and Child Health, and we hope that these findings will be beneficial to policy makers at a national level.

**Response to Reviewer #5 Comments:**

Author response: Initiating antenatal care (ANC) in the first trimester and identifying obstetric risk factors early are crucial for improving birth outcomes. Also, service providers’ knowledge of predictors of initiation of ANC has implications for obstetric risk management. This can help improve quality of care. However, English language needs some input; a few have provided.

General comments

1. The abstract convey what is in the manuscript

2. The data is sound.

3. The method section of the manuscript requires a major revision

4. The tables are genuine and well described

5. Some parts of the discussions needs revision to situate especially contrasting results in academic discourse

6. Conclusions reflect the results

I thus, recommend that the manuscript be accepted and the authors asked to revise the manuscript before publication.

Abstract

Word count =306

**R5.1: 1. The title: Towards stronger antenatal care: understanding predictors of late presentation to antenatal services and implications for obstetric risk management in Rwanda**

**The title needs revision to cover early initiation of ANC (<16 weeks) since it is part of the analysis as shown in Table 1 and the conclusion**

Author response: Thank you for this feedback. At the request of other reviewers, we have reframed our results to clarify our focus on late presentation to ANC, and no longer frame our results in terms of presenting to care before 16 weeks GA. As a result, we have kept the title as it was original submitted.

**Introduction**

**R5.2: 1. P. 5, line 89: insert ‘as’ for the sentence read such as the practice of disclosing pregnancy after the second trimester.**

Author response: Thank you catching this error. We have adjusted this wording in the revised version.

**R5.3: 2. P. 5, line 90-91: I suggest ‘poor experiences’ be replaced with ‘negative experiences’**

Author response: We have adjusted this wording in the revised version.

**R5.4: 3. P. 7, line 119-121: The authors should avoid repeating words sentences. The second ‘how’ in the sentence. I suggest: …, and inform messages tailored to multiparious and nulliparous women.**

Author response: We have adjusted this wording in the revised version.

**R5.5: 4. P. 7, 133: … completed ANC care… the ‘care’ is repetition and should be deleted.**

Author response: Thank you catching this error. We have adjusted this wording in the revised version.

**Results**

**R5.6: 5. P. 10, line 203 and 206-208: By conversion, a sentence should not begin with a figure. I suggest the sentences should be re-written.**

Author response: We have re-written this sentence in the revised version.

**R5.7: 6. P. 15 and 16, Tables 1, 2, 3 and 4 and 5: The ‘p-values’. The ‘p’ should be capitalised as ‘P-values’**

Author response: We have created new data tables based on suggestions from other reviewers, and all new tables have capitalized ‘P-values.’

**R5.8: 7. P. 16: Idetified is not necessary in Table 5**

Author response: We have re-written all of the table captions in our revised manuscript.

**R5.9: 8. All the information in brackets in Tables 1, 2, 3 and 4 (≥ 16 weeks GA) and very late (≥ 24 weeks GA) is not necessary. They are already difined in the text.**

Author response: Thank you for this suggestion. We have restructured our tables based on suggestions from other reviews and have this removed bracketed information in the revised draft.

**R5.10: 9. I expect the authors to emphasise finding regarding the benefits of initiating ANC < 16 weeks.**

Thank you for this feedback. We have re-emphasized the benefits of initiating ANC early in pregnancy in our conclusion.

**Discussion**

**R5.11: 10. P. 17, line 276: Despite ongoing to increase early ANC attendance, that the .... ‘that’ should be deleted.**

Author response: Thank you catching this error. We have adjusted this wording in the revised version.

**R5.12: 11. P. 17, 283-287: I suggest the authors look for evidence to explain and support their assertion that contribution to household income was a stronger predictor of delayed ANC-1. Also, could you please emphasise what your study adds?**

Author response: We have expanded upon this discussion in lines 356-362 and have added additional references. We now discuss that our findings stand in contrast to some of the existing literature regarding earning income and timing of ANC-1 initiation.

**R5.13: 12. P. 18, line 295: More effective strategy increase .... There should be ‘to’ as: ... strategy to increase ...**

Author response: Thank you catching this error. We have adjusted this wording in the revised version.

**R5.14: 13. P. 18, line 300-302: However, barriers to continuing ANC care may emerge for women for whom ANC-1. The sentence should be: However, barriers to continuing ANC care may emerge for women for whom ANC-1. I suggest ‘ANC’ be deleted and ‘for whom’ replaced with ‘whose’**

Author response: Thank for this suggestion. We have adjusted this wording in our revised manuscript.

**R5.15: 14. P. 19, line 333: why put ‘none’ into brackets. The sentence clear without ‘none’**

Author response: We have removed this from our revised draft, per your suggestions.

**R5.16: 15. P. 20, line 342: Despite conducting data strengthening activities, our investigation found lower than expected rates of obstetric risk factors were identified in the study population overall. ‘were identified’ is a repetition so need to be deleted.**

Author response: Thank you for catching this error. We have corrected in in our revised draft.

**R5.17: 16. P. 20, line 345-346: The sentence needs to be re-written to make comprehension easy for readers. I suggest: Other important screening results including diabetes and syphilis were systematically missing from the health record and thus were excluded from our final analysis.**

Author response: Thank you for this suggestion. We have updated our manuscript accordingly.

**R5.18: 17. P. 20, line 351: ... function of ANC care. ‘care’ is repetition and should be deleted.**

Author response: Thank you for catching this error. We have deleted this repeated word in the revised version.

**R5.19: 18. P. 20, line 357: Delete the second ‘in’ in the sentence. i.e. This study also reported large gaps in provider knowledge, including the ability to …**

Author response: We have deleted this word in the revised version.

**R5.20: Conclusion**

**19. The conclusion should also emphasise the benefits and predictors of early ANC attendance to inform interventions.**

Author response: We have re-written our conclusion, and include statements emphasizing the importance of early ANC attendance to facilitate identification and management of obstetric risk factors. We also emphasize that the results of this study provide insight into the populations at greatest risk of delaying ANC and may inform maternal health engagement strategies at a national level.

**R5.21: 20. The conclusion early intervention is too unspecific as this vary in different cultures**

Author response: Thank you for this feedback. We have re-written our conclusion in an attempt to make our recommendations more specific.

**R5.22: 21. The recommendation that community-based pregnancy testing could increase early pregnancy discovery and prompt earlier attendance at ANC clinics can be contested as the data does not show that late attendance is attributed to not being aware**

Author response: Thank you for this feedback. We have qualified our interpretation of these results in our discussion session in lines 318-328.

**Journal Requirements:**

**1. Please ensure that your manuscript meets PLOS ONE's style requirements, including those for file naming.**

Author response: We have updated our manuscript in accordance to PLOS ONE’s style requirements. Please see our revised manuscript.

**2. You indicated that you had ethical approval for your study. In your Methods section, please ensure you have also stated whether you obtained consent from parents or guardians of the minors included in the study or whether the research ethics committee or IRB specifically waived the need for their consent.**

Author response: We have added additional language clarifying consent for minors in our study: “The Rwandan National Ethics Committee and the University of California, San Francisco Institutional Review Board waived parental consent requirements for pregnant minors ages 15 and older.”

**3. Please include additional information regarding the survey or questionnaire used in the study and ensure that you have provided sufficient details that others could replicate the analyses. For instance, if you developed a questionnaire as part of this study and it is not under a copyright more restrictive than CC-BY, please include a copy, in both the original language and English, as Supporting Information.**

Author response: We have included this requested information as Supporting Information.

**4. Please ensure you have included the registration number for the clinical trial referenced in the manuscript.**

Author response: We have added the registration number for our clinical trial in the body of our manuscript.

**5. We note that you have stated that you will provide repository information for your data at acceptance. Should your manuscript be accepted for publication, we will hold it until you provide the relevant accession numbers or DOIs necessary to access your data. If you wish to make changes to your Data Availability statement, please describe these changes in your cover letter and we will update your Data Availability statement to reflect the information you provide.**

Author response:  Data files are available at the following DOI: 10.17605/OSF.IO/NKABY
