# Supplementary material for: Towards stronger antenatal care: Understanding predictors of late presentation to antenatal services and implications for obstetric risk management in Rwanda
Source: PLoS One. 2021 Aug 25;16(8):e0256415. doi: 10.1371/journal.pone.0256415 (PMC8386859; doi:10.1371/journal.pone.0256415)
Supplement: Attachment — Submitted filename: Comment.docx [file pone.0256415.s005.docx]

I appreciate the authors' effort because I have observed a significant improvement they made into this paper at this stage. The authors have also addressed the majority of my concern during their revision. But, still, I am not convinced in some cases.

1. For instance, regarding cutoff for “late” presentation, you referenced the cutoff used by the Rwandan Ministry of Health which is not standard or consistent with the cutoff used elsewhere. When I see published articles on this issue, the majority of articles published since 2018 used >12 weeks GA as “late” presentation. My concern here is that how yours compared with results obtained by this cutoff (>12 weeks GA)? In Rwanda, it may be OK.
2. Regarding the selection of measures of association, I am not clear with the reason why did you prefer reporting p-value than odds ratio? As you know p-value conveys a little information regarding the strength and direction of association compared with odds ratio. Additionally, even your p-value report is not consistent throughout. For instance, p<0.05, p<0.001, p=0.02, p=0.03,etc. When you report the p-value, report its exact value.
