# Supplementary material for: Towards stronger antenatal care: Understanding predictors of late presentation to antenatal services and implications for obstetric risk management in Rwanda
Source: PLoS One. 2021 Aug 25;16(8):e0256415. doi: 10.1371/journal.pone.0256415 (PMC8386859; doi:10.1371/journal.pone.0256415)
Supplement: Attachment — Submitted filename: Response to Reviewers.docx [file pone.0256415.s006.docx]

May 14th, 2021

Dr. Adeniyi Francis Fagbamigbe

Academic Editor, PLOS ONE

Dear Dr. Fagbamigbe,

Thank you for the opportunity to revise and resubmit our manuscript PONE-D-20-22930-R1, entitled “Towards stronger antenatal care: Understanding predictors of late presentation to antenatal services and implications for obstetric risk management in Rwanda”

We appreciate the reviewers’ additional comments and have considered and responded to the suggestions raised through the second review process. At the end of this letter, we have included a response to reviewers in which we address each comment the reviewers made. In our response to reviewers, the reviewers’ comments are numbered, and our responses follow below prefaced by “Author response.” Corresponding changes are highlighted in the manuscript text in the revised file.

Thank you again for your consideration of our manuscript with additional revisions. We look forward to hearing from you in due time regarding our submission and to respond to any further questions and comments you may have.

Sincerely,

Christina Schmidt (corresponding author)

Doctor of Medicine Candidate, School of Medicine

University of California San Francisco

Email: [christina.schmidt@ucsf.edu](mailto:christina.schmidt@ucsf.edu)

Phone: +1 503 703 4953

**Response to reviewer #1:**

*Major Revision:*

**R1.1** Tables 1 and 3: In the logistic model each characteristic will have only one p-value associated with it. In other words, p-values for each level of a characteristic should not be provided. Consider consulting a qualified statistician for assistance with reporting the results from logistic regression modeling.

Author response: Thank you for this feedback. We have updated Table 1 and report the associated OR, CI and p-values for each characteristic (i.e. age), in addition to each level of a characteristic (i.e. 15-19, 20-24, etc.). We have maintained the odds ratios and confidence intervals for each level at the request of another reviewer.

For Table 3, we report the p-value for each variable level, as we ran our logistic regression using categorical, dummy variables. Based on recommendations of our team’s statistician, we decided to run our analysis categorically so that we would be able to select clinically relevant categories for easier interpretation. For example, the risk of pregnancy-related complications increases after 35 years of age, and thus we collapsed all deliveries with a maternal age greater than 35 into one age category.

We did re-run our analyses using age, parity and educational attainment continuously, with the associated odd ratios, confidence intervals, and p-values for those variables:

16 weeks – Age (OR = 0.98, 95% CI:0.96,0.99, p = 0.001), parity (OR = 1.20, 95% CI: 1.15,1.25, p<0.001), educational attainment (OR = 0.85, 95% CI: 0.79,0.92, p<0.001)

24 weeks – Age (OR = 0.97, 95% CI: 0.96, 0.98, p<0.001), parity (OR = 1.23, 95% CI: 1.18,1.28, p<0.001), educational attainment (OR = 0.74, 95% CI: 0.67,0.83, p<0.001)

However, we prefer to keep these variables as categorical, as originally submitted, to facilitate easier application of these results to interventions seeking to improve ANC attendance.

*Minor Revisions:*
**R1.2** Express p-values more precisely than “p <0.05” and "p<0.01".

Author response: We appreciate this feedback. We have updated our manuscript to report our p-values more precisely throughout.

**R1.3** Line 228: Clarify the meaning of alpha=0.05.

Author response: We have removed this in our revised draft.

**R1.4** The standard statistical term for average is mean.

Author response: Thank you for this feedback. We have updated our manuscript and have replaced the word “average” with “mean.”

**R1.5** Line 246: Provide a measure of dispersion for the mean of GA at presentation.

Author response: Certainly. We have provided a standard deviation in the results section of our updated draft.

**R1.6** Clarify if the results presented in Tables 4 and 5 are based on univariate or multivariate models.

Author response: We have updated the titles of our tables to clarify that univariate models were used to produce these results.

**R1.7** Spell out a number when it occurs at the beginning of a sentence.

Author response: We have updated our manuscript accordingly. Thank you for this feedback.

**Response to reviewer #3:**

I appreciate the authors' effort because I have observed a significant improvement they made into this paper at this stage. The authors have also addressed the majority of my concern during their revision. But, still, I am not convinced in some cases.

**R2.1** For instance, regarding cutoff for “late” presentation, you referenced the cutoff used by the Rwandan Ministry of Health which is not standard or consistent with the cutoff used elsewhere. When I see published articles on this issue, the majority of articles published since 2018 used >12 weeks GA as “late” presentation. My concern here is that how yours compared with results obtained by this cutoff (>12 weeks GA)? In Rwanda, it may be OK.

Author response: Thank you for these thoughtful comments. We initially considered using >12 weeks GA as our definition of late presentation, but ultimately decided to use >16 weeks GA in our analysis. While we appreciate that other studies use GA >12 weeks to define late presentation to care, we believe that our work will have more locally relevant policy implications by using a GA cutoff of 16 weeks. During the conceptualization of our study, we discussed our proposed analysis with members of the Rwandan Ministry of Health’s Maternal, Child and Community Health Division, who expressed interest in conducting the present analysis due the potential for our results to shape the national approach to reaching the Ministry of Health’s performance targets. The national reporting tools in Rwanda report on presentation to ANC prior to 16 weeks, and this metric is used in the national Results Based Financing program. Further, the Rwanda DHS uses the same metric, recorded as months of pregnancy<4 at entry to care in the DHS 14-15, the latest DHS available at the time of this study. Ultimately, we believe that using 16 weeks as our cutoff will best facilitate the application of our findings to interventions targeting our study population.

**R2.2** Regarding the selection of measures of association, I am not clear with the reason why did you prefer reporting p-value than odds ratio? As you know p-value conveys a little information regarding the strength and direction of association compared with odds ratio. Additionally, even your p-value report is not consistent throughout. For instance, p<0.05, p<0.001, p=0.02, p=0.03, etc. When you report the p-value, report its exact value.

Author response: We appreciate this feedback and have updated our manuscript to provide odds ratios with associated confidence intervals, where applicable. This includes the results from Tables 2 through 5. Of note, we still report p-values in the body of our manuscript for Table 1, as the purpose of these models was variable selection. We have also updated the p-values throughout our revised manuscript to report exact values consistently.

**Response to reviewer #5:**

The manuscript is much improved, the text flows much better and almost all the issues raised have been addressed and acceptable for publication.

However, there are a few spelling and grammar mistakes (eg missing words, incorrect verb conjugation, missing articles etc) Examples

**R3.1** P.3 line 42, by conversion a sentence should not begin with a figure

Author response: We have updated our abstract appropriately.

**R3.2** P.5 line 83, ‘greater’ not necessary in the sentence.

Author response: We have removed this word in our revised manuscript.

**R3.3** P.6 line 101, "of" is missing in …parity and history of uncomplicated …

Author response: Thank you for catching this mistake. We have revised appropriately.

**R3.4** P23 line 437 the sentence should start with ‘The’ and replace the second ‘CHW’ with ‘their’ to avoid repetition.

Author response: We have edited the language of our manuscript to in response to this suggestion.

**R3.5** P23 line 441 replace ‘demonstrated’ with ‘demonstrate’

Author response: We have made the appropriate replacement.

**R3.6** P.22 line 402-404
This sentence seem to belittle the WHO’s call for more ANC visits. The eight ANC visit required is expected to ensure that all the necessary education activities and screening is carried out more thoroughly. Though arguably the intention is to ensure that even if a pregnant woman misses some ANC appointments, at least she will benefit from the education activities and screening than having four with some deciding to go only once or twice.

Author response: We have edited this paragraph to emphasize the importance of improving educational activities and screenings conducted at ANC visits, regardless of whether Rwanda stays with a four-visit system, or moves to an eight-visit system:

“…While Rwanda has a stronger healthcare system than many of its regional peers, our data show there is room for improvement in terms of capturing important risk factors in the antenatal period. A few studies have demonstrated that increasing the number of ANC visits a woman attends does not necessarily result in improved pregnancy outcomes, if important screening and educational activities are missed [37,38]. If Rwanda moves towards an eight-visit system, using additional contacts as an opportunity to enhance the delivery of educational activities and screening through provider trainings and standardized protocols will be important for improving maternal and neonatal outcomes.”

**R3.7** P.23 line 442-445.
You did not assess quality of care at ANC clinics. Why let your concluding statement be on quality of care. As stated above I suggest that being the last sentence, it should rather echo WHOs’ call for increasing number of ANC visits and the CHW outreach programme used to vigorously sensitise pregnant women and communities on the need to initiate ANC in the first trimester. This is likely to increase the number of ANC visits and improve pregnancy and birth outcomes as shown by your results.

Author response: We appreciate this feedback and have combined our final two paragraphs and have rephrased these sentences to read:

“Our results also demonstrated gaps in the identification and documentation of important obstetric risk factors at ANC-1 visits. Health centers should focus on enhancing the delivery of educational activities and improving antenatal screening through standardized provider trainings and protocols.”
